# The effect of stretch–shortening magnitude and muscle–tendon unit length on performance enhancement in a stretch–shortening cycle

**DOI:** 10.1038/s41598-021-94046-2

**Published:** 2021-07-16

**Authors:** Martin Groeber, Savvas Stafilidis, Arnold Baca

**Affiliations:** Department of Biomechanics, Kinesiology and Computer Science in Sport, Centre for Sport Science and University Sports, University of Vienna, Vienna, Austria

**Keywords:** Physiology, Bone quality and biomechanics

## Abstract

Stretch-induced residual force enhancement (rFE) is associated with increased performance in a stretch–shortening cycle (SSC). Although the influence of different range of motions and muscle–tendon unit lengths has been investigated in pure stretch-hold experiments in vivo, the contribution to a SSC movement in human muscles remains unclear. In two sessions, 25 healthy participants performed isometric reference (ISO), shortening hold (SHO) and SSC contractions on an isokinetic dynamometer. We measured the net knee-joint torque, rotational mechanical work, knee kinematics and fascicle behavior (m. vastus lateralis) of the upper right leg. In session 1 the SHO- and SSC-magnitude was changed respectively (SHO: 50°–20°, 80°–20° and 110°–20°; SSC: 20°–50°–20°, 20°–80°–20° and 20°–110°–20°) and in session 2 the muscle–tendon unit length (SHO: 50°–20°, 80°–50° and 110°–80°; SSC: 20°–50°–20°, 50°–80°–50° and 80°–110°–80°; straight leg = 0°). In both sessions, rotational work was significantly (*p* < 0.05) increased in the SSC compared to the SHO contractions (in the range of 8.1–17.9%). No significant difference of joint torque was found in the steady-state for all SSC-magnitudes compared to the corresponding SHO contractions in session 1. In session 2, we found only significantly (*p* < 0.05) less depressed joint torque in the SSC at the longest muscle–tendon unit length compared to the corresponding SHO condition, without any differences in knee kinematics and fascicle behavior. Therefore, the physiological relevance of rFE might be particularly important for movements at greater muscle–tendon unit lengths.

## Introduction

A stretch–shortening cycle (SSC) is a common muscle action during exercise and everyday movement. The SSC is defined as a stretching of the muscle–tendon unit prior to a shortening^1^. It has long been identified that this leads to increased force, torque, mechanical work and power during the shortening phase of the SSC compared to a pure shortening contraction, which is not preceded by active stretching (“SSC-effect”)^2,3^. Despite clear evidence that this phenomenon exists, the associated mechanisms are heavily debated in the literature as none of the mechanisms can entirely explain this SSC-effect^4–6^. The mechanisms attributed to this effect are the pre-activation of the muscle^7^, the stretch-reflex^8^ and the release of stored passive-elastic energy in the tendinous tissue^9,10^. Since it was shown that the SSC-effect was also visible in studies not related to the previously mentioned mechanism^6^ and the SSC-effect was present on the fiber level (meaning no serial-elastic component such as aponeurosis and tendon)^11^, another mechanism must also play a role in the SSC performance enhancement. The contribution of such an additional mechanism to the SSC performance enhancement should be found within the sarcomere^5,6,11–13^.

In stretch-hold experiments, the force or torque during active stretch but also in the isometric hold phase after active stretch is enhanced compared to a fixed-end reference contraction. This applies when the reference contraction is length-matched and has the same activation level. In literature, the enhancement is described to have a velocity-dependent^14,15^ transient force enhancement (FE) throughout the stretch phase^16^. This is characterized by a steep increase in force in the early stretch phase, followed by a slower rise^17,18^. The peak force during stretch is greater with increasing stretch velocity^18^. Followed by a long-lasting component, which is called residual force enhancement (rFE)^19–21^. It is thought that the protein titin mediates the phenomena of rFE^22,23^. In recent studies, it is proposed that the SSC-effect is also associated with rFE^6,12,24–26^.

The steady-state force or torque is decreased after shortening-hold (SHO) experiments compared to an isometric hold phase of a fixed-end contraction at the same muscle length and activation level. In literature this is called residual force depression (rFD)^19^. The key mechanism attributed to rFD is the stress-induced inhibition of the actin-myosin overlap zone^19,27,28^.

Stretch-hold or shortening-hold experiments do not mirror movements with real everyday significance. To ensure that these history-dependent properties of rFE and rFD are relevant for everyday human movement, controlled in vivo studies^29^ investigating SSC muscle action are required. In the context of a SSC, transient FE is increased due to elastic as well as viscoelastic structures^17,30^. Most of the energy storage cannot be explained by cross-bridge mechanism. This was showed in stretch experiments with isolated muscle fibers^17,30^ where in the presence of myosin inhibitors the peak non cross-bridge contribution and rFE remain high. Other structures as titin may store and release elastic energy during the stretch and the subsequent shortening phase of the SSC and thereby increasing the SSC-effect^5^.

In stretch-hold or shortening-hold, it was found that the amount of rFE and rFD could be influenced by various factors. In vitro studies show increased transient FE and rFE with higher stretch magnitude^15,20,31,32^. In vivo measurements show that rFE only depends on stretch magnitude in some circumstances (dependent on the muscle of interest)^33–35^. At shortening contractions, rFD increases with the work (force × shortening magnitude) performed during shortening in in vivo studies^36–38^.

Additionally, the history dependent properties are also dependent on the location of the muscle action on the force–length relationship. Greater transient FE and rFE occur at long muscle lengths compared to short muscle lengths for in vitro and in situ studies^31,39,40^. In addition to that, a significant larger SSC-effect was reported at relatively long muscle lengths in skinned fibers obtained from rabbit soleus^41^. These results are partially confirmed in in vivo experiments, where mostly increased rFE was found at longer muscle lengths in single joint movements^29,42–44^. However, another study testing the elbow flexor did not show rFE at long or short muscle lengths^45^. Considering rFD, previous studies revealed that the amount of rFD is related to the extent of force development during shortening^38,46^, which should mean that rFD should be larger at muscle lengths where more force can be produced due to the force–length relationship. However, no effect of muscle architecture^47^ and angular position^48^ on rFD during plantar flexion could be found. The operating length of muscle fascicles determine the force potential of the muscle, therefore it is crucial to measure fascicle behavior to identify if different contraction conditions are comparable. In contrast to the general conception that muscle–tendon unit change is accompanied by major changes in fascicle length, the m. vastus lateralis fascicles act mainly isometrically during the stance phase of running and walking for example^49^.

Based on the literature findings, we sought to investigate the influence of different SSC-magnitudes and muscle–tendon unit lengths on a SSC muscle action of the m. quadriceps femoris in humans. Firstly, we hypothesized that with greater SSC-magnitude transient FE (greater torque at the end of stretch) is enhanced in a SSC compared to an isometric pre-activation of a shortening contraction, which also influences the SSC-effect. But due to greater shortening range at greater SSC-magnitudes, we hypothesized that rFE is eliminated by the shortening phase which will result in a lack of differences in the steady-state isometric torque after the dynamic phase. And secondly, we expected no difference in transient FE^29^, but increased rFE at greater muscle–tendon unit lengths due to increased titin stiffness at longer muscle–tendon unit lengths^23,50^.

## Materials and methods

### Participants

30 healthy adults were recruited for this study. Five subjects did not finish the test protocol or data was missing. Finally, data from 25 participants were obtained (13 male and 12 female adults; age 26.2 ± 6.0 years; body mass 71.5 ± 11.7 kg; height 175.3 ± 8.9 cm). All participants took part in the study voluntarily and provided free written informed consent prior to the study. The participants had no injury to the right leg, neuromuscular disorders or cardiovascular problems. Eligibility for the study was decided with the help of an anamnesis questionnaire to detect the risk factors for physical activity. The experimental protocol was approved by the Ethics Committee of the University of Vienna (Reference Number: 00364).

### Experimental setup

The setup was configured similarly to a previous study^51^. Experiments were performed with an isokinetic dynamometer (HUMAC Norm Model 770; CSMi, Stoughton, MA, USA) to measure net knee-joint torque of the right leg in a sitting position. The analog signals of the dynamometer were captured with a Vicon Nexus A/D card (16bit) with a sampling frequency of 2 kHz. The seat of the isokinetic dynamometer was precisely adjusted to each participant, so that the rotation axis of the dynamometer was aligned with the lateral femoral condyles. The upper body was tightly fixed to the seat of the dynamometer by inextensible straps.

Kinematic data was recorded synchronously with nine cameras (Vantage V8, Vicon Nexus motion capturing system, Oxford, United Kingdom, 100 Hz). The measured torque was corrected by means of the kinematic data, since tissue deformation and dynamometer padding can result in a misalignment of the joint and dynamometer axes of rotation. Overall, eight reflective surface markers were used. Five were placed at the right leg (medial and lateral malleolus, the most prominent point of the lateral and medial femoral condyles and the trochanter major). Additionally, one marker was placed at the dynamometer´s rotation axis and two markers were placed on the dynamometer´s arm (one of them at the point of force application to define the distance between the dynamometer´s rotation axis and the line of action of the applied extension force).

To ensure a constant muscle contraction, two self-adhering surface electrodes (5 × 5 cm) were placed on the m. vastus lateralis proximal motor point and m. vastus medialis distal motor point to evoke constant muscle contraction using electrical stimulation (Digitimer DS8R, United Kingdom). The muscle motor points were detected by scanning the surface of the skin with a motor point pen (COMPEX, United Kingdom)^52^. Current was increased (100 μs square-wave pulses at 100 Hz) until the evoked tetanic torque reached 35% of maximal voluntary contraction (MVC) at a dynamometer angle of 20° (full knee extension was defined as 0°). The reached current was used for the whole experimental protocol. For the electrostimulation, we used an intensity of 35% of the MVC based on a previous study, which examined the history-dependent properties on the m. quadriceps femoris^51^. For that purpose, the current for the m. vastus lateralis and m. vastus medialis was adjusted together.

Fascicle behavior should be well controlled for the examination of the history-dependent properties^36^. Despite identical joint angles, fascicle length changes can be different^29^. Thus, fascicle length, pennation angle and fascicle length changes were determined using ultrasonography (Telemed ArtUS EXT-1H, IT, 70 Hz, Vilnius, Lithuania, EU). A linear probe (LV8-5N60-A2, field of view 60 mm) was tightened to the mid-belly of the m. vastus lateralis^53^ with a custom-made bracket. To synchronize the ultrasound data with the kinematic and dynamometer´s data, an analog signal (0–3 V) was generated when the ultrasound video was started and stopped, and it was captured by the Vicon system.

To synchronize all data, the analog ultrasound signal was used.

### Experimental protocol

**Figure 1:**
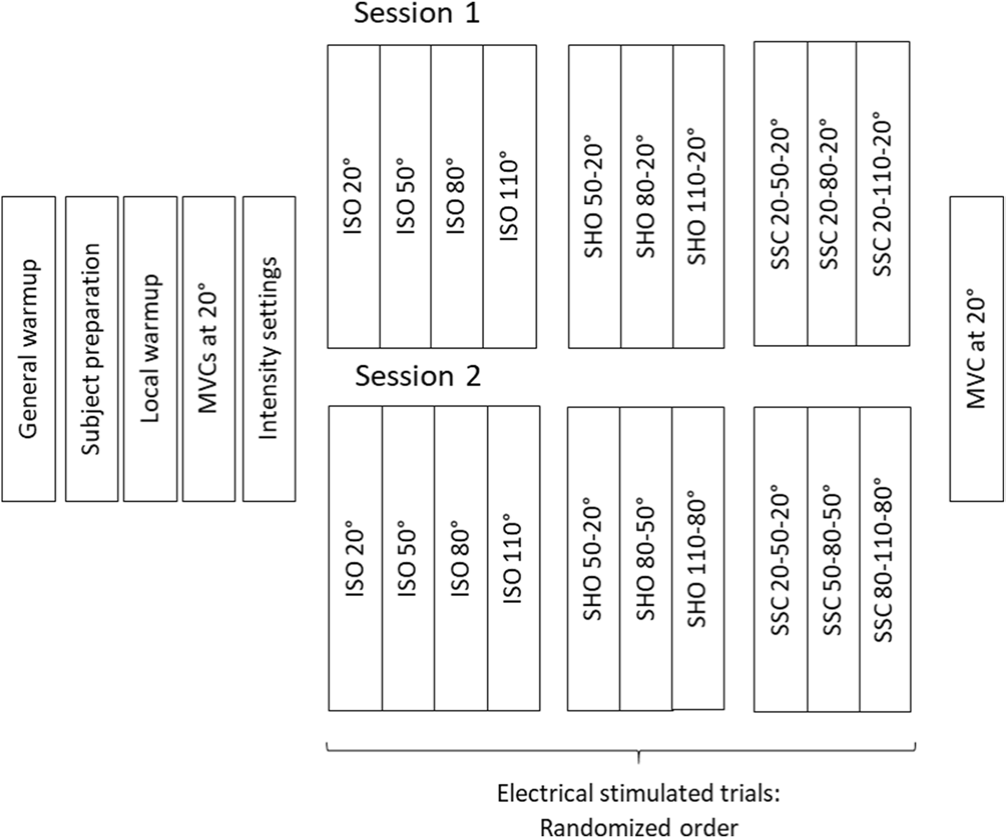
Experimental protocol. Each subject participated in two test sessions. Both test sessions comprised isometric fixed-end reference contractions (ISO), shortening-hold contractions (SHO) and stretch–shortening contractions (SSC). In session 1 the SHO- and SSC-magnitude and in session 2 the muscle–tendon unit length was modified.

All participants were familiar with the test setup. The study presented here consisted of two test sessions. Both started with a general warm-up at a bicycle ergometer (5 min, between 80 and 90 W), followed by a specific warm-up of the right leg on the isokinetic dynamometer (5 min, several submaximal contractions). Afterwards, the participants performed two MVCs at a dynamometer angle of 20°. During the MVCs verbal encouragement was provided and the highest developed torque was used for further analysis. Both test sessions comprised electrical stimulated isometric (ISO) at 20°, 50°, 80° and 110° dynamometer angle, shortening-hold (SHO) and stretch–shortening (SSC) muscle actions with different SHO- and SSC-magnitudes (session 1: SHO: 50°–20°, 80°–20° and 110°–20°; SSC: 20°–50°–20°, 20°–80°–20° and 20°–110°–20°) and different muscle–tendon unit lengths (session 2: SHO: 50°–20°, 80°–50° and 110°–80°; SSC: 20°–50°–20°, 50°–80°–50° and 80°–110°–80°) in randomized order (Fig. 1). Each muscle contraction was performed two times. Strictly speaking, the isometric contractions are fixed-end contractions, where muscle shortening can appear even when the joint angle is constant^54^. All ISO, SHO and SSC trials were submaximal, electrically stimulated contractions at 35% of MVC and the angular velocity of the dynamometer was always fixed to 60°/s. All dynamic muscle actions had an isometric pre-activation phase (until a plateau was reached) and an isometric steady-state hold phase for several seconds (3–5 s) after the knee rotation. All ISO, SHO and SSC contractions had a resting phase of two minutes in between to prevent fatigue^47^ and to check for fatigue appearance the participants were required to perform a final MVC contraction at 20°.

### Data processing

**Figure 2:**
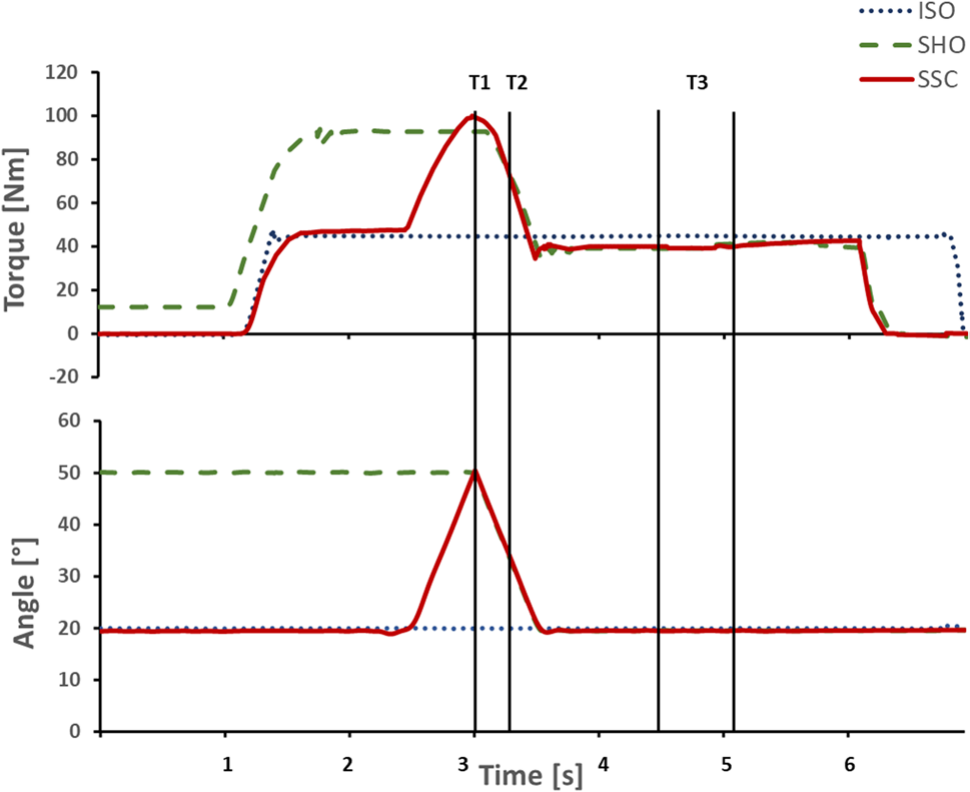
Exemplary representation of torque-time and dynamometer angle-time traces. The dotted blue line represents ISO, the dashed green line SHO and the continuous red line SSC. The vertical lines indicate the timepoint of data analysis, with T1 being end of stretch, T2 midpoint of shortening and T3 the steady-state interval where the mean torque was calculated.

The data of the isokinetic dynamometer was filtered with a zero-delay fourth-order Butterworth filter with a cut-off frequency of 10 Hz. Torque values were corrected for gravity for all participants. For each condition the mean values were used for statistical analysis. The different conditions were compared at different instances. At T1, which is defined as the end of stretch in the SSC condition (50°, 80° or 110° dynamometer angle depending on the trial). At T2 which is defined as the midpoint of the shortening range and T3 in the isometric steady-state. At T3, ISO, SHO and SSC condition were compared 1–1.5 s after the knee rotation, while here the average values of the 0.5 s interval window were used (Fig. 2). At T3 all contraction for each participant were visually inspected to assure, that the contractions reached a steady-state.

External rotational work during the shortening was calculated as the integral of knee-joint torque using a numerical trapezoidal method.

1 $$W = \smallint M d\varphi = \smallint M \cdot \omega dt$$

with W as the mechanical work during the shortening phase [J], M as torque [Nm] and ω as rotational velocity [rad/s].

Kinematic data was further processed with a zero-delay fourth-order Butterworth filter with a cutoff frequency of 10 Hz. The knee-joint angles of the different conditions were compared at the previously described instances, because there can be a discrepancy between the measured knee angle and dynamometer-defined angle at higher contraction intensities^51^. Additionally, the kinematic data was used to implement a previously suggested inverse dynamic approach^55^. The measured knee joint torque can deviate from the resultant joint torque up to 4.3%^56^. Due to these differences in the torque measured by the dynamometer and the resultant joint torque, we addressed this shortcoming and implemented the previously mentioned inverse dynamic approach. These differences are triggered by a shift of knee joint axis due to tissue deformation and the compliance of the dynamometer^55^. With this approach, the movement of the segment relative to the dynamometer arm can be corrected. Resultant joint torque was calculated as follows:

2 $$M_{res} = M_{Meas} \cdot \frac{{d_{K} }}{{d_{B} }}.$$

with M_res_ as the resultant joint torque, M_Meas_ the measured torque, d_B_ as the lever arm of the applied force to the dynamometer axis and d_K_ as the lever arm of force to the knee joint^55^.

The pennation angle was defined as the angle between muscle fascicle and deep aponeusis, and the fascicle length as the distance between the intersection of the muscle fascicle to the deep and superficial aponeurosis. Fascicle length changes during shortening was defined as the difference of fascicle length at T1 and T3. The average values of three measurements were used for further analysis. In the event that the whole muscle fascicle was not visible on the ultrasound image, a linear continuation was assumed. To measure the non-visible part, trigonometry was used assuming an error of less than 2.4%^57,58^. It was reported that fascicle length values are reliable within the same session, however between sessions the extrapolation method errors cannot be predicted^59^. In our study, the fascicle length was only compared within the same session. Additionally, to test the interrater reliability, two evaluators processed the ultrasound images.

### Statistical analysis

All data were normally distributed (Shapiro–Wilk test) and therefore a paired t-test with dependent variables or repeated measures ANOVA was used. T-test was used to compare the MVCs at the beginning and at the end of the test session and for the comparison of the same conditions at the two different sessions. Two-way ANOVA (within-within subject design) was used to examine the interaction (session 1: condition × magnitude, session 2: condition × muscle–tendon unit length) on mechanical work, knee joint torque, knee angle, pennation angle, fascicle length and fascicle length change. If sphericity was violated, Greenhouse–Geisser correction was used. In the case of significant interaction, subsequent post hoc comparisons with Bonferroni adjustments were executed to compare the conditions (ISO, SHO and SSC) at each rotation magnitude (session 1) and muscle–tendon unit length (session 2). The effect size was calculated with partial eta squared (η^2^). Fascicle length and pennation angle measurements were examined for interrater reliability by using the intraclass correlation coefficient (ICC, two-way mixed model, single measures).

### Ethics approval

Approval was obtained from the ethics committee of the University of Vienna (Reference Number: 00364). The procedures used in this study adhere to the tenets of the Declaration of Helsinki.

## Results

### Initial conditions

With the applied current, a mean isometric joint torque of 32.6 ± 11.2% at session 1 and 31.5 ± 6.6% at session 2 of MVC was achieved at 20° dynamometer angle. Additionally, t-tests revealed no significant differences (*p* > 0.05) between the MVC at the beginning of the test session (session 1: 97.5 ± 25.8 Nm and session 2: 100.5 ± 34.0 Nm) compared to the end of the test session (session 1: 97.0 ± 25.4 Nm and session 2: 101.4 ± 33.5 Nm) and no difference between the sessions (*p* > 0.05), indicating no fatigue and similar conditions at both sessions.

### Joint torque and work measurements

**Figure 3:**
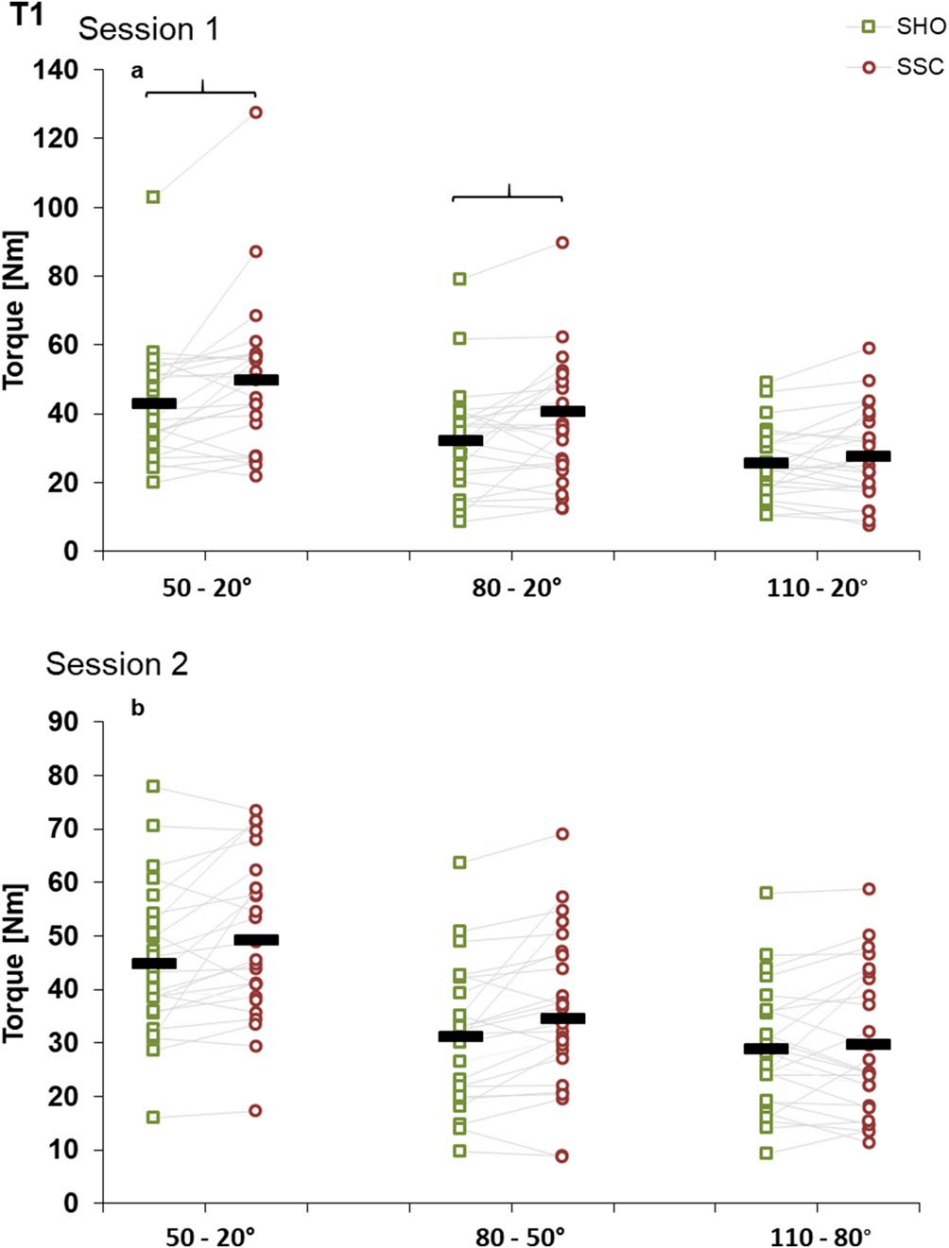
Mean (n = 25, thick horizontal line) (and mean of each individual subject) values of joint torque in session 1 with varying SHO- and SSC-magnitudes (**a**) and in session 2 with varying muscle–tendon unit length (**b**) at the onset of shortening (T1). Green squares represent shortening-hold conditions (SHO), whereas red circles represent stretch–shortening conditions (SSC). In the SSC condition the shortening phase was preceded by the corresponding stretch phase. Braces indicate significant (*p* < 0.05) differences between SSC and the corresponding SHO condition.

At T1 in session 1, two-way ANOVA showed a significant interaction (condition × magnitude) on knee joint torque (*p* = 0.037, η^2^ = 0.124). Main effect of condition showed increased torque for the SSC compared to SHO condition (*p* = 0.009, η^2^ = 0.240). Post-hoc comparison revealed increased torque at the end of stretch (T1) in the SSC 20°–50°–20° (*p* = 0.008) and 20°–80°–20° (*p* = 0.014) compared to the isometric pre-activation in the SHO condition; no statistical difference could be found after the stretch in the SSC 20°–110°–20° (*p* = 0.252) (Fig. 3a). This resulted in a 15.4 ± 15.8% and 24.4 ± 17.4% FE, for the SSC conditions at 20°–50°–20° and 20°–80°–20°, respectively. The relative increase at T1 for the SSC 20°–110°–20° was 8.2 ± 16.8% (not significant).

**Figure 4:**
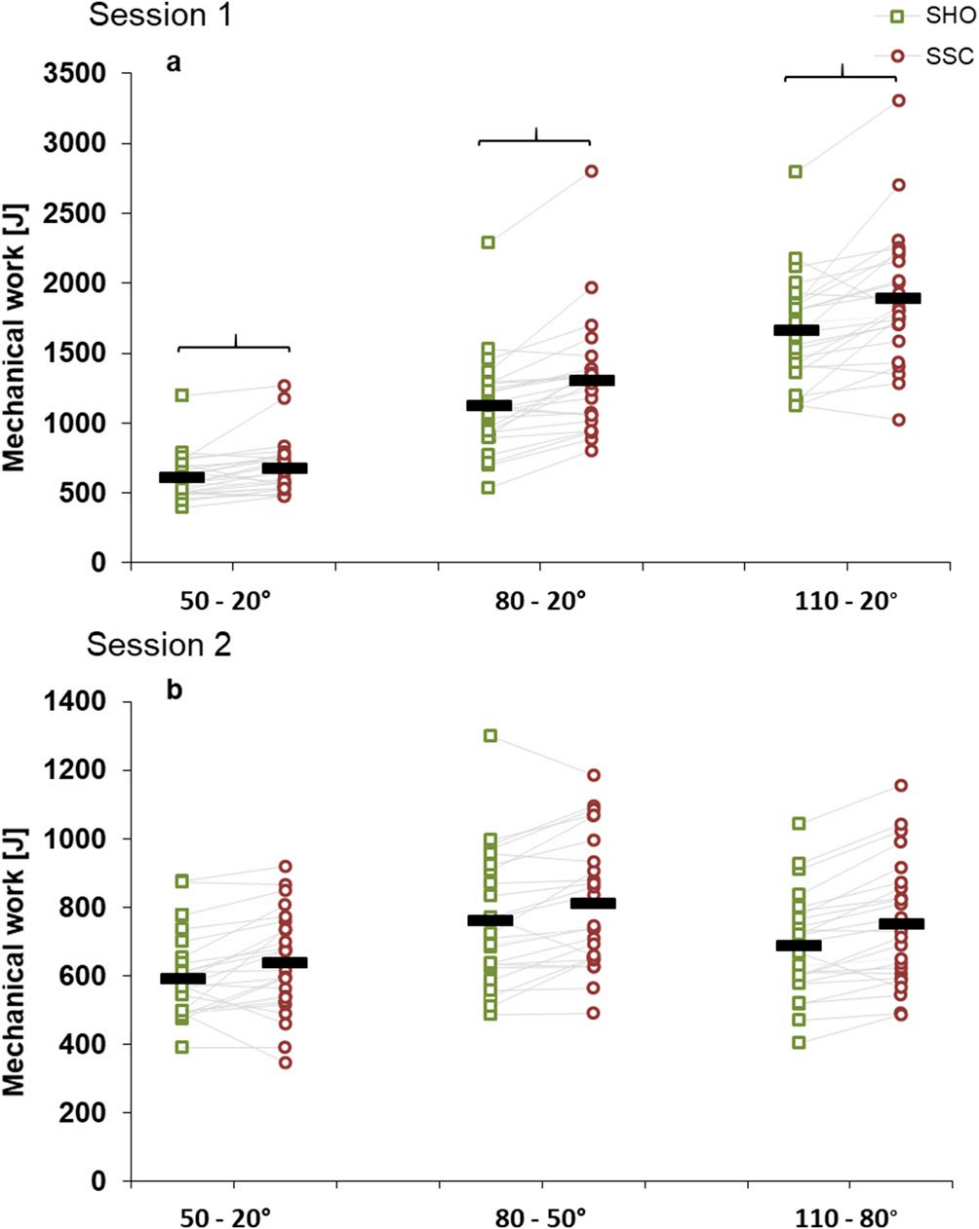
Mean (n = 25, thick horizontal line) (and mean of each individual subject) values of mechanical work during shortening in session 1 with varying SHO- and SSC-magnitudes (**a**) and session 2 with varying muscle–tendon unit lengths (**b**). Green squares represent shortening-hold conditions (SHO), whereas red circles represent stretch–shortening conditions (SSC). In the SSC condition the shortening phase was preceded by the corresponding stretch phase. Braces indicate significant (*p* < 0.05) differences between SSC and the corresponding SHO condition.

In session 1, significant interaction (condition × magnitude) was revealed for mechanical work (*p* < 0.001, η^2^ = 0.267). Main effect of rotation magnitude (*p* < 0.001, η^2^ = 0.925) and condition (*p* < 0.001, η^2^ = 0.524) showed increased mechanical work with higher rotation magnitude and for the SSC condition. At all rotation magnitudes, mechanical work was significantly greater during the shortening in the SSC compared to the corresponding SHO condition (50°–20°: *p* = 0.003, 80°–20°: *p* < 0.001, 110°–20°: *p* < 0.001) (Fig. 4a). The percentage increases of mechanical work in the SSCs (SSC-effect) were 11.1 ± 15.1, 17.9 ± 16.2 and 14.0 ± 16.0% compared to SHO contraction for the ranges 50°–20°, 80°–20° and 110°–20°, respectively.

**Figure 5:**
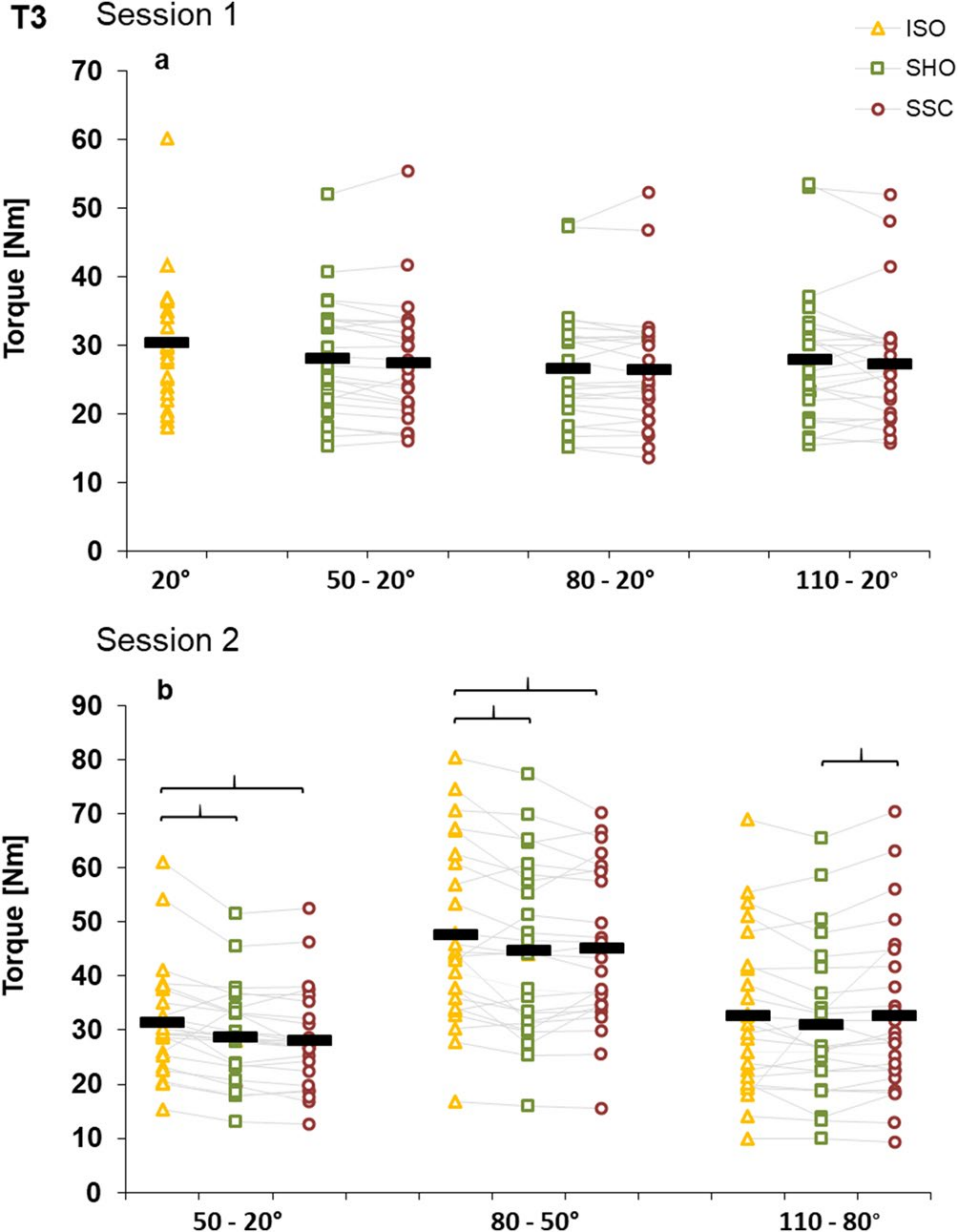
Mean (n = 25, thick horizontal line) (and mean of each individual subject) values of torque in the steady-state after knee rotation (T3) in session 1 with varying SHO- and SSC-magnitudes (**a**) and in session 2 with varying muscle–tendon unit lengths (**b**). Yellow triangles represent the isometric fixed-end reference conditions at corresponding dynamometer angle (ISO), green squares represent shortening-hold conditions (SHO), whereas red circles represent stretch–shortening conditions (SSC). In the SSC condition the shortening phase was preceded by the corresponding stretch phase.

At T3, in the isometric steady-state no significant interaction (condition × magnitude) was found (*p* = 0.134, η^2^ = 0.071) is session 1. Main effect of condition revealed significant results (*p* < 0.001, η^2^ = 0.527). Compared to the fixed-end reference condition we found a significant torque depression for SHO (*p* < 0.001) and SSC (*p* < 0.001) and no difference was found between SHO and SSC condition (*p* = 0.296). The main effect of magnitude was significant (*p* = 0.035, η^2^ = 0.126); with increased rotation magnitude we found lower torque at T3, meaning higher torque depression. Compared to the fixed-end reference contraction (ISO) the steady-state torque was depressed in SHO and SSC conditions (SHO: 50°–20°: 7.9 ± 8.0%, 80°–20°: 12.6 ± 8.1%, 110°–20°: 13.8 ± 10.9%; SSC: 20°–50°–20°: 10.0 ± 10.5%, 20°–80°–20°: 13.1 ± 12.6%, 20°–110°–20°: 15.4 ± 11.9%) (Fig. 5a).

A two-way ANOVA showed no significant interaction (condition × muscle–tendon unit length) on the joint torque at T1 (*p* = 0.174, η^2^ = 0.70) in session 2. The main effect of condition showed significantly higher torque values at T1 for the SSC condition compared to the SHO condition (*p* = 0.008, η^2^ = 0.257). The main effect for muscle–tendon unit length was also significant (*p* < 0.001, η^2^ = 0.714) (Fig. 3b). The percentage increase in the SSC conditions 20°–50°–20°, 50°–80°–50° and 80°–110°–80° were 9.7 ± 12.3, 10.9 ± 12.9 and 6.9 ± 15.2%, respectively (no significant interaction).

For mechanical work in session 2, no significant interaction (condition × muscle–tendon unit length) was found (*p* = 0.544, η^2^ = 0.25). The main effect for condition (*p* < 0.001, η^2^ = 0.435) and muscle–tendon unit length (*p* < 0.001, η^2^ = 0.408) showed significantly higher mechanical work with greater muscle–tendon unit length and for the SSC condition (Fig. 4b). The percentage increase of mechanical work (SSC-effect) was almost identical at all different muscle–tendon unit lengths (50°–20°: 8.1 ± 15.3%, 80°–50°: 8.5 ± 7.4% and 110°–80°: 8.6 ± 7.8%).

In the steady–state (T3), a significant interaction (condition × muscle–tendon unit length) (*p* = 0.033, η^2^ = 0.127) was identified in session 2. The main effect of muscle–tendon unit length was significant (*p* < 0.001, η^2^ = 0.636), as well as the main effect for condition (*p* < 0.001, η^2^ = 0.296). Further comparison with Bonferroni post hoc correction showed significant higher torque for the SSC condition compared with the SHO condition at the greatest muscle–tendon unit length (*p* = 0.043). No statistical difference was found between SHO: 50°–20° and SSC: 20°–50°–20° (*p* > 0.05) and between SHO: 80°–50° and SSC: 50°–80°–50° (*p* > 0.05). Compared to the fixed-end reference contraction torque was significantly depressed (*p* < 0.05), except for the SSC 80°–110°–80° and the SHO 110°–80° condition (SHO: 50°–20°: 8.1 ± 11.0%, 80°–50°: 6.0 ± 9.9%, 110°–80°: 4.9 ± 9.3%; SSC: 20°–50°–20°: 10.0 ± 13.6%, 50°–80°–50°: 5.3 ± 8.7%, 80°–110°–80°: 0 ± 8.8%) (Fig. 5b).

Comparing the same conditions in session 1 and 2 revealed no differences between the sessions (T1: SHO: 50°–20°, *p* = 0.626; SSC: 20°–50°–20°, *p* = 0.206; mechanical work: SHO: 50°–20°, *p* = 0.420; SSC: 20°–50°–20°, *p* = 0.168; T3: SHO: 50°–20°, *p* = 0.585; SSC: 20°–50°–20°, *p* = 0.594).

### Knee joint angle

**Figure 6:**
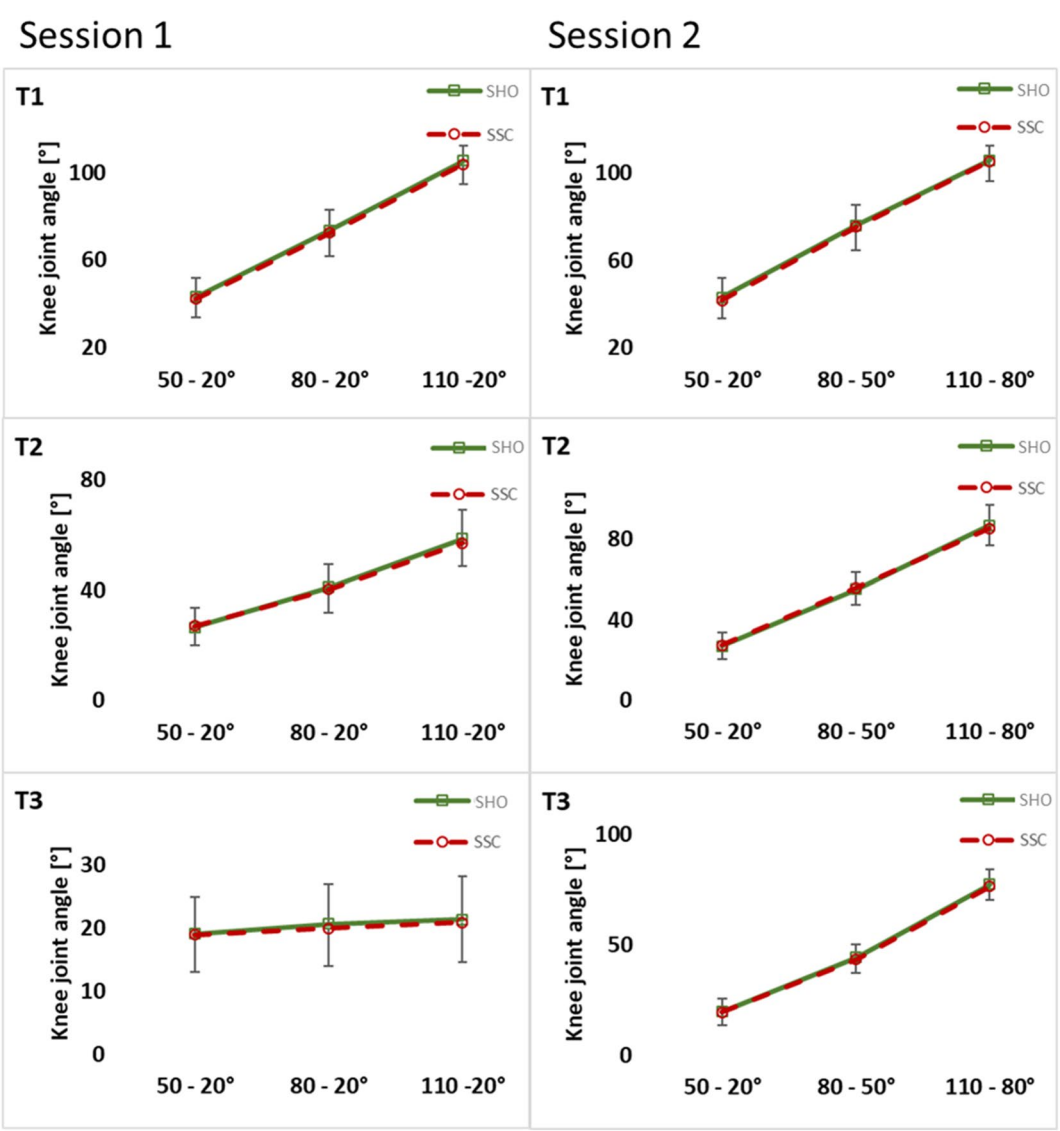
Mean (± SD, n = 25) values of knee-joint angle in both test sessions. T1 is the time point at the onset of shortening, T2 in the middle of the shortening phase, T3 the time point at steady-state after the dynamic phase. No significant interaction (condition × magnitude) and (condition × muscle–tendon unit length) was found at all instances (T1–T3).

No significant interaction (condition × magnitude) could be found at all instances for the knee joint angle (T1: *p* = 0.843, η^2^ = 0.007; T2: *p* = 0.314, η^2^ = 0.043; T3: *p* = 0.771, η^2^ = 0.010) in session 1. Main effect for condition showed no difference of knee joint angle between SHO and SSC condition (T1: *p* = 0.083, η^2^ = 0.115; T2: *p* = 0.494, η^2^ = 0.019; T3: *p* = 0.215, η^2^ = 0.061) (Fig. 6).

For knee joint angle at session 2, no significant interaction (condition × muscle–tendon unit length) was found at all instances (T1: *p* = 0.771, η^2^ = 0.011; T2: *p* = 0.629 η^2^ = 0.019; T3: *p* = 0.418, η^2^ = 0.036). We did not find a significant main effect of condition on knee-joint angle (T1: *p* = 0.062, η^2^ = 0.137; T2: *p* = 0.070, η^2^ = 0.130; T3: *p* = 0.109, η^2^ = 0.103) (Fig. 6).

### Fascicle behavior

Two independent investigators analyzed fascicle length and pennation angle. For fascicle length, mean ICC across participants for all measurements was 0.87 (ranging from 0.84 to 0.89) and for pennation angle 0.86 (ranging from 0.84 to 0.89). These results indicate a good interrater reliability^60^.

**Figure 7:**
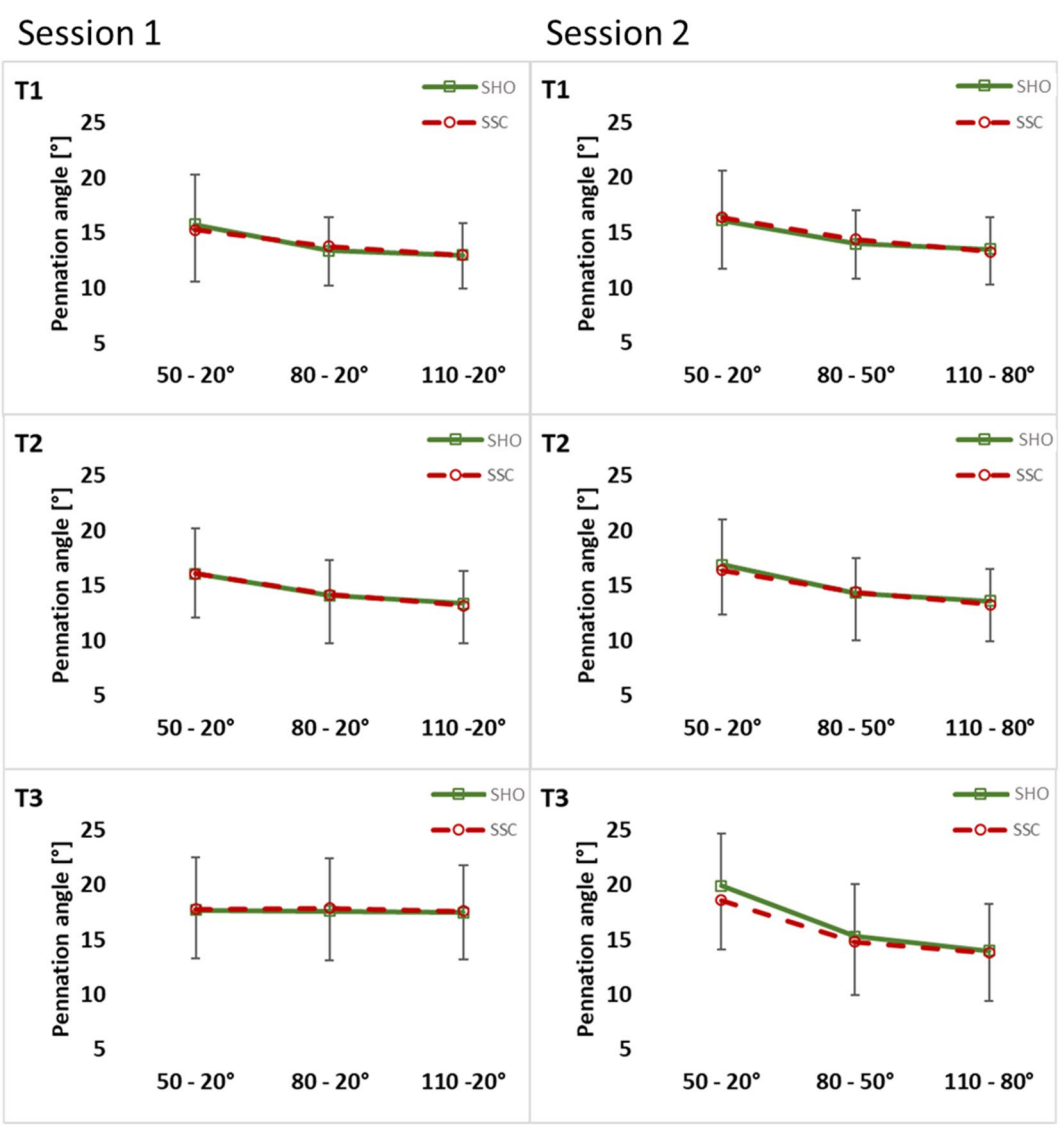
Mean (± SD, n = 25) values of pennation angle in both test sessions. T1 is the time point at the onset of shortening, T2 in the middle of the shortening phase, T3 the time point at steady-state after the dynamic phase. No significant interaction (condition × magnitude) and (condition × muscle–tendon unit length) was found at all instances (T1–T3).

**Figure 8:**
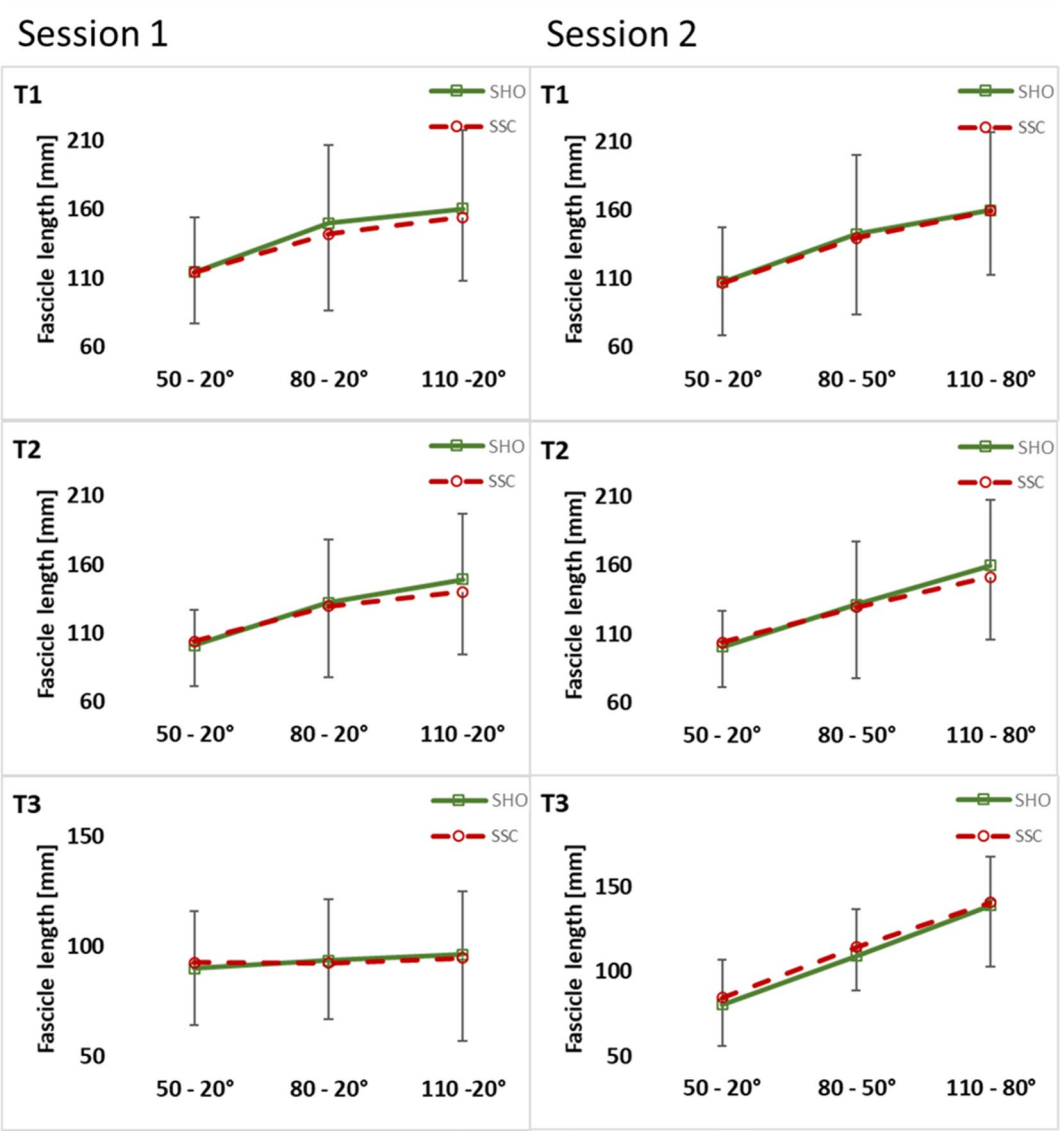
Mean (± SD, n = 25) values of fascicle length in both test sessions. T1 is the time point at the onset of shortening, T2 in the middle of the shortening phase, T3 the time point at steady-state after the dynamic phase. No significant interaction (condition × magnitude) and (condition × muscle–tendon unit length) was found at all instances (T1–T3).

In session 1, statistical analysis of pennation angle revealed no significant interaction (condition × magnitude) at T1 (*p* = 0.187, η^2^ = 0.070), T2 (*p* = 0.942, η^2^ = 0.002) and T3 (*p* = 0.201, η^2^ = 0.067) (Fig. 7) and for fascicle length at T1 (*p* = 0.489, η^2^ = 0.035), T2 (*p* = 0.244, η^2^ = 0.065) and T3 (*p* = 0.247, η^2^ = 0.067) (Fig. 8). No significant interaction could be found for fascicle length changes (*p* = 0.849, η^2^ = 0.003).

Further, the main effect of condition showed no significant difference between the SHO and SSC condition for pennation angle (T1: *p* = 0.704, η^2^ = 0.006; T2: *p* = 0.979, η^2^ = 0.001; T3: *p* = 0.408, η^2^ = 0.030) and for fascicle length (T1: *p* = 0.625, η^2^ = 0.012; T2: *p* = 0.100, η^2^ = 0.123; T3: *p* = 0.741, η^2^ = 0.006) is session 1. Main effect of condition showed also no difference between SHO and SSC condition for fascicle length change (*p* = 0.846, η^2^ = 0.002).

In session 2, no significant interaction (condition × muscle–tendon unit length) was found for pennation angle (T1: *p* = 0.053, η^2^ = 0.132; T2: *p* = 0.381, η^2^ = 0.043; T3: *p* = 0.054, η^2^ = 0.136) (Fig. 7) and fascicle length (T1: *p* = 0.981, η^2^ = 0.001; T2: *p* = 0.558, η^2^ = 0.026; T3: *p* = 0.469, η^2^ = 0.033) at all instances (Fig. 8). No significant interaction could be found for fascicle length changes (*p* = 0.913, η^2^ = 0.003).

Also the main effect for condition revealed no difference between SHO and SSC for pennation angle (T1: *p* = 0.088, η^2^ = 0.132; T2: *p* = 0.417, η^2^ = 0.030; T3: *p* = 0.082, η^2^ = 0.143) and fascicle length (T1: *p* = 0.843, η^2^ = 0.002; T2: *p* = 0.928, η^2^ = 0.001; T3: *p* = 0.326, η^2^ = 0.048) is session 2. Main effect of condition also showed no difference between SHO and SSC condition for fascicle length change (*p* = 0.900, η^2^ = 0.001).

## Discussion

The main purpose of this study was to examine the influence of SSC-magnitude and muscle–tendon unit length on the SSC performance, with a special focus on the contribution of the history-dependent properties of muscle contraction. The contribution of rFE was independent of the magnitude, but significantly less rFD was found after the SSC compared to the corresponding pure shortening contraction at the longest muscle–tendon unit length. As expected, the average rotational work values during the concentric phase were significantly greater for all SSCs compared to the corresponding shortening contraction (SHO) at all rotation magnitudes. However, no difference in the SSC-effect with increased rotation magnitude or at different muscle–tendon unit lengths was demonstrated. For all instances (T1–T3) and in both sessions, we could not find any differences for the control variables fascicle length, pennation angle, fascicle length change and knee joint angle, meaning the different contraction conditions are very well comparable with each other in our study. In our study, the conditions SHO 50°–20° and SSC 20°–50°–20° were the same in session 1 and 2. The individual comparison by means of t-test revealed no significant differences between the sessions at all instances. This result shows a good repeatability of this experiment, as the identical SHO and SSC conditions, which took place on a different day, showed no differences.

### Effect of stretch–shortening magnitude

Similarly to other studies^6,43^, increased joint torque at the end of stretch (T1) compared to the isometric pre-activation of the SHO condition was found in session 1. However, contrary to our expectations, we could only find significant transient FE for the smaller SSC-magnitudes and not after the greatest stretch in the SSC 20°–110°–20° condition (FE of 8.2%, not significant) (Fig. 3a). This is in contrast to in vitro studies, which showed higher transient FE with increasing stretch magnitude^15^. In vivo studies reported different results. For example, during or at the end of stretch, the transient FE of adductor pollicis was increased with greater rotation^35^, whereas no difference was reported for larger muscle groups of the lower extremities^33,34^. One explanation of these discrepancies could be the presence of some inhibitory effects during voluntary movement with larger muscle stretches^34^. Similarly to voluntary contractions, in the present study with electrical stimulation we also found no increased transient FE with higher stretch magnitude. One possible explanation of our results could be that at T1 the lowest torque values were observed at 110°, and transient FE seems to be greater at higher absolute force/torque values^51,61^.

The absolute values of mechanical work during shortening increased with greater rotation magnitude (Fig. 4a). Contrary to what we expected, the SSC-effect did not increase with larger rotation magnitude. We expected that due to greater stretch, more elastic energy could be stored and later released by the tendon and increased passive forces would rise caused by the protein titin, which acts in a spring-like manner^62^. The SSC mechanism of different activation dynamics and stretch-reflex activity can be neglected in our study, since we had electro-stimulated contractions and an isometric pre-activation. At the greatest rotation magnitude, the torque at T1 was not statistically different between SHO and SSC, thus it is reasonable to expect that the mechanical work would also be similar. However, relative to the differences at the initial condition (T1), the percentage increase of the mechanical work at the SSC condition 20°–110°–20° compared to the corresponding SHO condition is greater than at the other rotation magnitudes; thus indicating a more “effective” use of the stored elastic energy. Hence, although the enhanced absolute force properties might be eliminated by the subsequent shortening^63^, we possibly observe a relative increase in the mechanical work due to a more “effective” use of stored elastic energy.

In contrast to a positive influence of elastic recoil, stretch-induced force-enhancing effects in the contractile element should be visible in the steady-state after the dynamic phase (T3, Fig. 5a)^6^. At T3, we did not find significant interaction, but the main effect of condition revealed significant torque depression for both SHO and SSC compared to the ISO contraction. Torque depression was between 7.9 and 15.4% in session 1, which is within the range of other quadriceps femoris studies^64–66^. Additionally, the main effect for rotation magnitude (Fig. 5a) illustrated higher torque depression with greater rotation magnitude. These observations have also been made in shortening-hold experiments; the shortening causes conformational changes to the structure of the actin filament which can inhibit cross bridge attachments and reducing muscle stiffness^67^. Overall, the influence of the rotation magnitude on the history-dependent properties in a SSC shows the same increase of rFD with greater shortening as in SHO contractions. This confirms the assumption that rFE is only stretch-magnitude dependent under specific circumstances, which depend on the muscle of interest^29^. Such a difference might be inhibited by the amount of muscle–tendon compliance^33^. In our experiment all contractions ended at small muscle–tendon unit lengths although most studies investigating rFE at m. quadriceps femoris used relative long muscle–tendon unit lengths^33,68^. The results might be different, when testing different SSC-magnitudes only at long muscle–tendon unit lengths, with a final muscle length at the plateau region or the descending limb of the force–length relationship. However, in human movement the m. quadriceps femoris mostly has a relative short muscle–tendon unit length and based on the results of single muscle fibers rFE should be visible on the ascending limb of the force–length relationship^69^.

### Effect of muscle–tendon unit length

At T1, we did not find a significant interaction (condition × muscle–tendon unit length). We expected to have greater transient FE at longer muscle–tendon unit lengths due to a reduction in myofilament lattice spacing^70^ and increased titin stiffness at longer muscle lengths^23^, and inappropriate cross-bridge attachments at shorter muscle–tendon unit lengths^39^. These assumptions could not be confirmed in our study but our findings agree with a previous study, where the authors suggested that at purely eccentric contractions the transient FE is not muscle-length dependent^29^.

Mechanical work during the shortening in the SSCs was significantly higher compared to the SHO conditions in session 2. The SSC-effect was almost constant at all muscle–tendon unit lengths (8.1–8.6%). This result does not correspond to a study in skinned muscle fibers, where a greater SSC-effect was reported at long muscle lengths^41^. As previously stated, two mechanisms most likely can explain the performance enhancement in the SSC found in our study. It is known from the literature that the tendon elongation should increase dependent on the applied force^71^. Since in our experiment we reached the highest torque values at the smaller angular position at T1 (Fig. 3b), more elastic energy should therefore be stored and released in these conditions. In contrast to this mechanism, a contribution of a rFE related mechanism should also be visible in the steady-state (T3)^6^. It was speculated that the contribution of rFE is greater at longer muscle–tendon unit lengths due to greater titin stiffness^23^. We found significant rFD compared to the ISO condition at the smaller angular positions, with no difference between the SHO and SSC condition. The only difference between SHO and SSC was found at the greatest muscle–tendon unit length, whereas significant higher torque values were found for the SSC condition, without any significant differences in pennation angle, fascicle length or fascicle length changes during shortening. This indicates that rFE related mechanisms are responsible for this difference between SHO and SSC at the longest muscle–tendon unit length. This can also be supported by in vivo stretch-hold experiments, which showed that rFE is muscle–tendon unit length dependent^29,44^. Additionally, another study observed rFE at short and long muscle lengths, and also noticed greater rFE at longer muscle lengths^43^. Analogous to the previous stretch experiments, which observed torque values above the fixed-end reference contraction (rFE), we found evidence of rFE in the form of reduced rFD after the stretch–shortening cycle.

In literature, greater rFE at longer muscle length is associated with an increase in titin stiffness^50^. The contribution of such a titin engagement also seems to be greater in our in vivo experiment, but did not lead to any magnification of the SSC-effect.

### Limitations

The contribution of titin engagement is difficult to identify in in vivo experiments, since muscle tendon unit compliance might change with the muscle length^72^. However, in vitro experiments might give a better understanding of the history dependent effects, but for relevance in human movement such in vivo experiments are necessary^73^.

Participants performed a MVC at the beginning of the test session and at the end of the test session to check for fatigue appearance. This approach has its limitation in estimating the fatigue of the individual sub-muscles from MVC measurements of the whole four-headed muscle group. The relative contribution of the individual sub-muscles is different depending on the amount of the torque and the knee angle^74,75^ and the variability in MVC measurements can be large. However, the contractions were fully randomized (condition and stretch–shortening magnitude or muscle–tendon unit length), therefore fatigue would be a systematic error. Additionally, we stimulated with only 35% of MVC and had a resting phase of 2 min between the contractions, therefore it is reasonable to expect no fatigue appearance in our study as also shown by the pre-post MVC control test.

We used electrical stimulation to prevent neural inhibition, which might change depending on rotation magnitude and muscle–tendon unit length. In addition, an unresolved issue is still the potential neural contribution to non-responders to rFE^76^. However, one limitation is that the electrical stimulated contractions we have used in our experiment differ from voluntary contractions with their asynchronous and varied firing frequencies^64^. A previous study investigated the relative contribution of the sub-muscles of m. quadriceps femoris during voluntary activation^74^, however the relative contribution of quadriceps femoris constituents to knee extensor torque during electrical superficial stimulation remains unknown^77^. Another limitation, which concerns relevance during human locomotion, is the examination of a single joint movement where, in natural movements, we have voluntary multi-joint contractions. The motor points for the application of the electrodes for electrical stimulation were identified at a dynamometer angle of 20°. Through the rotation and the large range of motion, the activation might reduce at greater knee flexion angels due to the movement of the motor point relative to the electrodes. This can explain higher torque values at 50° dynamometer angle compared to the 80° dynamometer angle at T1 (Fig. 3). If activation would be constant, higher torque values would be expected at T1 for the SHO 80°–20° and SSC 20°–80°–20° (kinematic data revealed a knee joint angle of ~ 72°) compared to SHO 50°–20° and SSC 20°–50°–20°, where the measured knee joint angle was approximately 42°. The 72° should be close to plateau region and the 42° should be on the ascending limb of the torque–angle relationship. However, in different studies the greatest torque was also found at different angles in knee extension contractions in the range of 55°–90°^78^. Nevertheless, this is a systematic error and the SHO and SSC can be compared and hence a possible activation reduction at great knee flexion angles would be the same for both contraction conditions.

Ultrasound measurement of only m. vastus lateralis represents a limitation. The measurement only corresponds to the behavior of the individual muscle but not of the entire muscle group. It was reported that fascicle behavior can be different between m. vastus lateralis and the deeper m. vastus intermedius^79^, but the muscle architecture of one superficial muscle is similar to the architecture in another superficial muscle^80^. Thus, we assume in our experiment the measured architectural changes of m. vastus lateralis are also representative for the superficial stimulated m. vastus medialis.

## Conclusion

Overall, we observed a SSC-effect (enhanced mechanical work during the shortening phase) at all SSCs. The contribution of rFE was enhanced with longer muscle–tendon unit length (without a clear relative increase in mechanical work), whereas no influence of rotation magnitude was found. This result indicates that during SSCs in the knee extensors, the magnitude of the contribution of rFE is different depending on the muscle–tendon unit length. At shorter muscle–tendon unit lengths and at greater SSC-magnitudes, the stretch-induced force enhancing effects were attenuated by the subsequent shortening of the muscle. Therefore, the physiological relevance of rFE might be particularly important for movements at greater muscle–tendon unit lengths.

## Data Availability

The datasets generated during and/or analysed during the current study are available from the corresponding author on reasonable request.
